# Supplementary material for: Does a waiting room increase same-day treatment for sexually transmitted infections among pregnant women? A quality improvement study at South African primary healthcare facilities
Source: medRxiv. 2025 Feb 13:2025.02.12.25322145. Preprint. [Version 1] doi: 10.1101/2025.02.12.25322145 (PMC11844596; doi:10.1101/2025.02.12.25322145)
Supplement: Supplement 1 — Additional file 1: Additional file 1.docx, Socio-economic and health characteristics of included and excluded participants. Additional file 2: Additional file 2.docx, Percentage of women who waited for results by clinic and intervention date (14 December 2023). Additional file 3: Additional file 3.docx, Secondary analysis 1: All clinics, grouped by waiting room availability. Additional file 4: Additional file 4.docx, Additional file 4 – Sub-group analysis: Clinics A and B combined, grouped by presence of a sexually transmitted infection. [file media-1.pdf]

Additional files 1 to 5

Does a waiting room increase same-day treatment for sexually transmitted infections among pregnant women? A quality improvement study at South African primary healthcare facilities

Ranjana M.S. Gigi,[1,2] Mandisa M. Mdingi,[1] Lukas Bütikofer,[3] Chibuzor M. Babalola,[4] Jeffrey D. Klausner,[4] Andrew Medina-Marino,[5] Christina A. Muzny,[6] Christopher M. Taylor,[7] Janneke H.H.M. van de Wijgert,[8] Remco P.H. Peters,[1,9*] Nicola Low[2*]

1. Research Unit, Foundation for Professional Development, East London, South Africa
2. Institute of Social and Preventive Medicine, University of Bern, Bern, Switzerland
3. CTU Bern, Department of Clinical Research, University of Bern, Bern, Switzerland
4. Department of Population and Public Health Sciences, Keck School of Medicine, University of Southern California, Los Angeles, California, USA
5. Department of Psychiatry, Perelman School of Medicine, University of Pennsylvania, Philadelphia, Pennsylvania, USA
6. Division of Infectious Diseases, University of Alabama at Birmingham, Birmingham, Alabama, USA
7. Department of Microbiology, Immunology, and Parasitology, Louisiana State University Health Sciences, New Orleans, LA
8. Julius Center for Health Sciences and Primary Care, University Medical Center Utrecht, Utrecht University, Utrecht, Netherlands
9. Department of Medical Microbiology, University of Pretoria, Pretoria, South Africa

***** equal contribution

Corresponding author: Prof. Nicola Low (nicola.low@unibe.ch)

Additional files 1 to 5

Additional file 1 – Socio-economic and health characteristics of included and excluded participants

	Included (N=624)	Excluded (N=876)	P-value
**Age (in years)**			
Mean (SD)	27.6 (6.20)	29.0 (5.96)	<0.001
Median [Min, Max]	27.0 [18.0, 44.0]	29.0 [17.0, 44.0]	
**Gestational age (in weeks)**			
Mean (SD)	14.4 (5.86)	14.2 (5.82)	0.366
Median [Min, Max]	14.1 [3.43, 26.9]	13.7 [2.86, 26.9]	
Missing	0 (0%)	4 (0.5%)	
**Number of previous pregnancies**			
Mean (SD)	1.34 (1.27)	1.42 (1.29)	0.266
Median [Min, Max]	1.00 [0, 10.0]	1.00 [0, 7.00]	
**Education level**			
Less than Grade. 10	48 (7.7%)	55 (6.3%)	0.02
Grade 10 or 11	209 (33.5%)	271 (30.9%)	
Grade 12	310 (49.7%)	419 (47.8%)	
Diploma	38 (6.1%)	89 (10.2%)	
Degree	19 (3.0%)	41 (4.7%)	
Missing	0 (0%)	1 (0.1%)	
**Employment status**			
Not employed	383 (61.4%)	501 (57.2%)	0.116
Employed	241 (38.6%)	375 (42.8%)	
**Personal income (ZAR per month)**			
None	383 (61.4%)	505 (57.6%)	0.001
< 1000	31 (5.0%)	21 (2.4%)	
1001 - 5000	157 (25.2%)	249 (28.4%)	
5001 - 10'000	48 (7.7%)	75 (8.6%)	
> 10'000	5 (0.8%)	26 (3.0%)	
**Vaginal discharge (observed or reported)**			
Yes	82 (13.1%)	106 (12.1%)	0.602
No	542 (86.9%)	770 (87.9%)	
**HIV status**			
HIV negative	433 (69.4%)	615 (70.2%)	0.833
HIV positive on ART	158 (25.3%)	210 (24.0%)	
HIV positive, not on ART	33 (5.3%)	49 (5.6%)	
Missing	0 (0%)	2 (0.2%)	
**Time normally spent waiting to see a nurse/doctor (in minutes)**			
Mean (SD)	62.2 (55.6)	56.0 (50.3)	0.123
Median [Min, Max]	45.0 [0, 360]	45.0 [0, 420]	
**Travelling time to the clinic (in minutes)**			
Mean (SD)	25.4 (17.6)	23.9 (14.0)	0.797
Median [Min, Max]	20.0 [0, 120]	20.0 [1.00, 120]	
**Money spent to attend clinic (in ZAR)**			
Mean (SD)	37.4 (34.8)	39.2 (35.8)	0.651
Median [Min, Max]	34.0 [0, 300]	32.0 [0, 300]	

Additional files 1 to 5

Additional file 2 – Percentage of women who waited for results by clinic and intervention date (14 December 2023)

Waited for results, % (95% CI) n/N	Clinic A	Clinic B	Clinic C	Clinic D	Clinic E	Overall
	Intervention: waiting room introduced		No intervention: always had a waiting room		No intervention: never had a waiting room	
Pre-intervention	26 (16-39)<br>17/65	10 (4-18)<br>8/84	94 (86-98)<br>77/82	68 (43-86)<br>13/19	18 (12-26)<br>21/118	37 (32-42)<br>136/368
Post-intervention	18 (9-32)<br>9/50	2 (0-12)<br>1/49	80 (66-90)<br>41/51	69 (49-84)<br>20/29	23 (15-35)<br>18/77	35 (29-41)<br>89/256
Total	23 (16-32)<br>26/115	7 (3-13)<br>9/133	89 (82-93)<br>118/133	69 (54-81)<br>33/48	20 (15-26)<br>39/195	36 (32-40)<br>225/624

Additional file 3 – Secondary analysis 1: All clinics, grouped by waiting room availability

	Waiting room vs. no waiting room
Absolute percentage difference, % (95% confidence interval)	+39 (+31 to +47)
Adjusted absolute percentage difference, % (95% confidence interval)	
Adjusted for employment status of participants	+39 (+31 to +47)
+ STI positivity	+41 (+33 to +49)
+ proportions of symptomatic participants	+39 (+31 to +47)
+ load shedding (in 3 categories: no load shedding, stage 1-3, stage 4-6)	+42 (+35 to +50)
+ the distance in metres to the nearest food shop	+7 (–4 to +18)
+ work experience of the nurse (in 3 categories: 9 years=low, 15 years=middle, 33 years=long)	–8 (–15 to –0.3)

Additional files 1 to 5

Additional file 4 – Sub-group analysis: Clinics A and B combined, grouped by presence of an STI

	Percentage waited, % (95% CI, n/N)	Absolute percentage difference, % (95% CI)	Adjusted[1] absolute percentage difference, % (95% CI)
No STI	13 (7-21, 13/101)		
Pre-intervention	18 (7-35, 6/34)		
Post-intervention	10, (4-20, 7/70)	–9 (–26 to –7)	–11 (–29 to +7)
STI present	15 (10-22, 22/147)		
Pre-intervention	16 (10-24, 19/118)		
Post-intervention	10 (3-29, 3/29)	–6 (–20 to –8)	–5 (–23 to +12)
p-value for interaction between STI presence and absence		0.79	0.76

Legend: CI, 95% confidence interval; 1, adjusted for employment status of participants, STI positivity, proportions of symptomatic participants, load shedding (in three categories: no load shedding, stage 1-3, stage 4-6), and the distance in metres to the nearest food shop

Additional files 1 to 5

Additional file 5 – Reasons for not waiting for STI test results, or for changing intention, grouped by clinic and pre/post-intervention

	Clinic A	Clinic B	Clinic C	Clinic D	Clinic E	Overall
Total number of participants	115	133	133	48	195	624
Responded to questions about waiting for results, n (%)	74 (64)	77 (58)	76 (58)	40 (83)	125 (64)	392 (63)
**"Are you planning to wait for your results today?"**						
Yes	48 (65)	7 (9)	65 (86)	35 (88)	20 (16)	175 (45)
No	26 (35)	70 (91)	11 (15)	5 (13)	105 (84)	217 (55)

**"What is your main reason why you are not intending to wait today?"**	**Clinic A (n=26) n (%)**	**Clinic B (n=70) n (%)**	**Clinic C (n=11) n (%)**	**Clinic D (n=5) n (%)**	**Clinic E (n=105) n (%)**	**Overall (n=217) n (%)**
Have to get back to my kids/family	5 (19)	13 (19)	3 (27)	2 (40)	43 (41)	66 (30)
Have to get to work/school	8 (31)	18 (26)	5 (46)	1 (20)	28 (27)	60 (28)
Hungry	5 (19)	12 (17)	0	1 (20)	11 (11)	29 (13)
Want to go to the shop	0	9 (13)	1 (9)	0	16 (15)	26 (12)
Not feeling well	2 (8)	8 (11)	0	0	1 (1)	11 (5)
No time/going somewhere	0	5 (7)	1 (9)	0	2 (2)	8 (4)
Load shedding	3 (12)	2 (3)	0	0	0	5 (2)
Referred to hospital	1 (4)	1 (1)	0	0	2 (2)	4 (2)
Too hot	0	2 (3)	0	0	0	2 (1)
Boring	0	0	0	0	2 (2)	2 (1)
Transport availability	0	0	1 (9)	0	0	1 (1)
No space to wait	1 (4)	0	0	0	0	1 (1)
Didn't come alone to the clinic	0	0	0	1 (20)	0	1 (1)
Tired	1 (4)	0	0	0	0	1 (1)

**"What would make you change your mind?"**	**Clinic A (n=26) n (%)**	**Clinic B (n=70) n (%)**	**Clinic C (n=11) n (%)**	**Clinic D (n=5) n (%)**	**Clinic E (n=105) n (%)**	**Overall (n=217) n (%)**
Nothing	20 (77)	28 (40)	11 (100%)	3 (60)	95 (91)	157 (72)
If I did not have other commitments/not be in a hurry	2 (8)	23 (33)	0	0	1 (1)	26 (12)
Food	3 (12)	10 (14)	0	1 (20)	9 (9)	23 (11)
If I had someone looking after the kids	0	5 (7)	0	1 (20)	0	6 (3)
If I can feel better	0	3 (4)	0	0	0	3 (1)

Additional files 1 to 5

Comfortable waiting space	1 (4)	1 (1)	0	0	0	2 (1)
**“What made you change your mind about waiting for the results?”**	**Clinic A (n=1), n (%)**	**Clinic B (n=0), n (%)**	**Clinic C (n=1), n (%)**	**Clinic D (n=2), n (%)**	**Clinic E (n=7, n (%)**	**Overall (n=11) n (%)**
Waiting for other clinic procedures (for example medication)	1 (100)	0	0	0	6 (86)	7 (64)
Education about importance of waiting by the nurse	0	0	1 (100)	2 (100)	0	3 (27)
Returned to clinic after being called	0	0	0	0	1 (14)	1 (9)
